# Unpacking the intractability of childhood stunting: an introduction to the UKRI GCRF Action Against Stunting Hub

**DOI:** 10.1136/bmjpo-2023-002333

**Published:** 2024-03-22

**Authors:** Modou Lamin Jobarteh, Kaitlin Conway-Moore, Dinesh Yadav, Darius Testa Tata, Umi Fahmida, Babacar Faye, Bharati Kulkarni, Deepak Saxena, Claire Heffernan

**Affiliations:** 1 Epidemiology & Population Health, London School of Hygiene & Tropical Medicine, London, UK; 2 Health Services Research and Policy, London School of Hygiene & Tropical Medicine, London, UK; 3 Department of Population Health, London School of Hygiene & Tropical Medicine, London, UK; 4 Southeast Asian Ministry of Education Organisation Regional Centre for Food and Nutrition (SEAMEO RECFON), Central Jakarta, DKI Jakarta, Indonesia; 5 Faculté de médecine, Université Cheikh Anta Diop (UCAD), Dakar, Senegal; 6 National Institute of Nutrition, Hyderabad, India; 7 Indian Institute of Public Health Gandhinagar, Gandhinagar, India

**Keywords:** Growth, Epidemiology, Infant, Gastroenterology, Breastfeeding

Despite concerted efforts, the global community is off-track in its ambition to reduce the number of stunted children under 5 years by 40% by 2025.^1 2^ Stunting in children is thought to be a result of adversities in early life with multiple contributors including inadequate nutritional intake, environmental insults and intergenerational transmission of risk. While we know the pathways to stunting are numerous, our understanding of the convergence of these pathways remains a critical block. As such, childhood stunting can be described as a ‘mosaic’ where there is knowledge of the individual components, but inadequate understanding of the interactions or inter-relationships between the individual elements/drivers comprising the whole.^3^ In addition, the current definition of stunting is based on the measurement of child length, calculated as Length-for-Age-Z score (LAZ) 2 SDs below the median of the WHO growth standard. While this is important in adopting a universally accepted standard definition, the approach is rather too simplistic. The definition does not take into consideration the profoundly complex pathophysiology of stunting and its far-reaching consequences on the growth and development, future health and well-being of the affected children, communities and nations. Accordingly, research on stunting tends to focus on a single outcome or at best, parts of a system, with inadequate holistic exploration of underlying interconnections. Indeed, by defining childhood stunting as a single outcome, it may be argued that we have fostered a lack of understanding of the pathway each child has been on to reach such an outcome. The Action Against Stunting Hub (AASH), a partnership of 18 institutions, funded by the UK Research and Innovation’s Global Challenges Research Fund aims to generate a holistic understanding of the complex drivers of stunting and their interactions to better inform prevention and treatment strategies. This article introduces AASH, the related workstreams and interdisciplinary synergies.

The AASH’s approach to broadening the understanding of child stunting was inspired by ‘the development niche’ paradigm developed by Super and Harkness to investigate child development.^4^ According to the authors, the developmental niche is comprised of three core elements: (1) the physical and social environment underpinning a child’s daily life, (2) the dominant cultural norms regarding childcare/rearing and (3) the psychological make-up of parents and caretakers. The AASH then adapted the ‘developmental niche paradigm’ with the addition of a fourth element, the child’s basic biology and reframed it as the ‘bio-development’ niche. The bio-development niche is operationalised using ‘the Whole Child Approach’ (WCA). The WCA investigates the role of deep biology (epigenetics, gut health, neurocognition, nutrition, parasitology), the home environment, food environment, water, hygiene and sanitation, the educational and caregiving environment in childhood stunting. The WCA also elicits and clarifies shared values toward nutrition and stunting at the community level (figure 1). Thus, the WCA is used to investigate the complex underpinnings of childhood stunting, shifting focus from the disciplinary silos to building interdisciplinary connections. In this manner, we anticipate this approach will generate a holistic understanding of stunting and support the creation of appropriate tools such as decision support tools, predictive markers and therapeutic targets to address childhood stunting.

**Figure 1:**
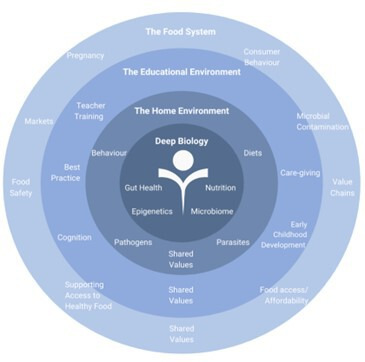
UK Research and Innovation’s Global Challenges Research Fund Action Against Stunting Hub ‘Whole Child Approach’ to investigate childhood stunting. A child-centric conceptual framework focusing on the ‘whole’ child, instead of the disparate drivers, provides a holistic understanding of the drivers and outcomes associated with stunting. The approach investigates the role of the home environment, food systems, dietary intake, water, hygiene and sanitation, shared values, neurocognition and education environments, gut health, microbiome, nutrition, parasitology and epigenetics in childhood stunting.

The WCA is investigated via a combination of observational and intervention studies. The observational study involves prospective pregnancy cohorts in India, Indonesia and Senegal, where women are recruited during pregnancy with mother–infant pairs followed longitudinally until 24 months post partum. The intervention trials test the efficacy of egg supplementation during pregnancy and early childhood on child growth and development in Indonesia and India, respectively. Additionally, the impact of synbiotic (a combination of prebiotics and probiotics) administration during the first 6 months of infancy on child growth and development is currently being assessed in a trial in Senegal. Furthermore, the COVID-19 pandemic and its disruptions to the global food supply, earnings and access to essential healthcare services is projected to increase the number of stunted children in low-income and middle-income countries by an estimated 22 million more children.^5^ The AASH is responding to these emerging exacerbating factors by setting up cohort studies in India to understand the impact of the pandemic and SARS-CoV-2 (severe acute respiratory syndrome – coronavirus 2) infection during pregnancy on food availability and intake, access to healthcare and health services, pregnancy outcomes, growth, and development in early childhood.

The workstreams informing the ‘whole child approach’ to stunting are epigenetics, gut health, nutrition, food and home environment, cognition and education and shared values. The detailed protocols underpinning each workstream are published in this BMJ Paediatrics Open Supplement. Briefly, the epigenetic workstream conducts analyses of both the wider and specific regions of the epigenome in DNA extracts from the saliva samples of fathers, mothers and infants enrolled in the study. The gut health workstream investigates the relationship between gastrointestinal health and childhood stunting with the aim to better understanding the timing, implications and contributions of enteric dysfunctions in childhood stunting. The nutrition workstream investigates the role of maternal nutrition, health and well-being, and IYCF (infant and young child feeding) on stunting. This workstream also investigates maternal stress, depression, psychosocial support on fetal and infant growth and development. The education and cognition workstream investigates the links between childhood stunting and cognition, caregiving and the wider educational environment. The workstream uses measures of ECED (early childhood education and development) to profile the early development of children. The food systems and home environment workstream investigates the contributions of food and home environment to childhood stunting. The shared value workstream explores community values regarding childhood stunting. Shared value is defined as implicit or explicit underlying sociocultural beliefs, concepts and principles. The workstream will investigate the societal beliefs and practices related to stunting and its drivers.

While unravelling the roles of these individual components is important, the interdisciplinary connections, interactions and synergies between the workstreams move the discourse beyond disciplinary boundaries into a novel, holistic understanding of the complex underpinnings of childhood stunting. The AASH is developing an interdisciplinary aid within its All-Hub Data Repository to support exploration of the interdisciplinary interactions. The repository also facilitates the capturing and curation of research data from the three countries into a central database with a range of bespoke tools including a decision support tool. Equally importantly, the research includes a MEL (monitoring, Eevaluation, and Llearning) toolkit to support monitoring of the field research activities, tracking its progress, milestones and share learning outcomes in (near) real-time.

In conclusion, the interdisciplinary research at AASH presents a new frontier aimed at moving the dial away from individual exposures to broadening the understanding of childhood stunting by carefully unpacking its critical predisposing factors and outcomes. In this manner, we believe the AASH will provide researchers, programme and policy developers with the knowledge and tools to guide global efforts in eradicating childhood stunting and its associated developmental delays.

## Supplementary Material

Reviewer comments

Author's manuscript
