# Supplementary material for: Unpacking the intractability of childhood stunting: an introduction to the UKRI GCRF Action Against Stunting Hub
Source: BMJ Paediatr Open. 2024 Mar 22;8(Suppl 1):e002333. doi: 10.1136/bmjpo-2023-002333 (PMC10961497; doi:10.1136/bmjpo-2023-002333)
Supplement: Author's manuscript [file bmjpo-2023-002333.draft_revisions.pdf]

BMJ Paediatrics Open

BMJ Paediatrics Open is committed to open peer review. As part of this commitment we make the peer review history of every article we publish publicly available.

When an article is published we post the peer reviewers' comments and the authors' responses online. We also post the versions of the paper that were used during peer review. These are the versions that the peer review comments apply to.

The versions of the paper that follow are the versions that were submitted during the peer review process. They are not the versions of record or the final published versions. They should not be cited or distributed as the published version of this manuscript.

BMJ Paediatrics Open is an open access journal and the full, final, typeset and author-corrected version of record of the manuscript is available on our site with no access controls, subscription charges or pay-per-view fees (http://bmjpaedsopen.bmj.com).

If you have any questions on BMJ Paediatrics Open's open peer review process please email info.bmjpo@bmj.com

BMJ Paediatrics Open

Unpacking the Intractability of Childhood Stunting: the UKRI GCRF Action Against Stunting Hub

Journal:	*BMJ Paediatrics Open*
Manuscript ID	bmjpo-2023-002333
Article Type:	Viewpoint
Date Submitted by the Author:	16-Oct-2023
Complete List of Authors:	Jobarteh, Modou; London School of Hygiene & Tropical Medicine, Epidemiology & Population Health<br>Conway-Moore, Kaitlin; London School of Hygiene & Tropical Medicine, Health Services Research and Policy<br>Yadav, Dinesh; London School of Hygiene & Tropical Medicine, Department of Population Health<br>Tata, Darius Testa; London School of Hygiene & Tropical Medicine, Department of Population Health<br>Fahmida, Umi; Southeast Asian Ministry of Education Organisation Regional Centre for Food and Nutrition (SEAMEO RECFON)<br>Faye, Babacar; Université Cheikh Anta Diop (UCAD), Faculté de médecine<br>Kulkarni, Bharati; National Institute of Nutrition<br>Saxena, Deepak ; Indian Institute of Public Health Gandhinagar<br>Heffernan, Claire; London School of Hygiene & Tropical Medicine, Department of Population Health
Keywords:	Growth, Epidemiology, Infant, Gastroenterology, Breastfeeding

SCHOLARONE™
Manuscripts

I, the Submitting Author has the right to grant and does grant on behalf of all authors of the Work (as defined in the below author licence), an exclusive licence and/or a non-exclusive licence for contributions from authors who are: i) UK Crown employees; ii) where BMJ has agreed a CC-BY licence shall apply, and/or iii) in accordance with the terms applicable for US Federal Government officers or employees acting as part of their official duties; on a worldwide, perpetual, irrevocable, royalty-free basis to BMJ Publishing Group Ltd ("BMJ") its licensees and where the relevant Journal is co-owned by BMJ to the co-owners of the Journal, to publish the Work in this journal and any other BMJ products and to exploit all rights, as set out in our licence.

The Submitting Author accepts and understands that any supply made under these terms is made by BMJ to the Submitting Author unless you are acting as an employee on behalf of your employer or a postgraduate student of an affiliated institution which is paying any applicable article publishing charge ("APC") for Open Access articles. Where the Submitting Author wishes to make the Work available on an Open Access basis (and intends to pay the relevant APC), the terms of reuse of such Open Access shall be governed by a Creative Commons licence – details of these licences and which Creative Commons licence will apply to this Work are set out in our licence referred to above.

Other than as permitted in any relevant BMJ Author's Self Archiving Policies, I confirm this Work has not been accepted for publication elsewhere, is not being considered for publication elsewhere and does not duplicate material already published. I confirm all authors consent to publication of this Work and authorise the granting of this licence.

Confidential: For Review Only

Unpacking the Intractability of Childhood Stunting: the UKRI GCRF Action Against Stunting Hub

Modou L. Jobarteh[1], Kaitlin Conway-Moore[2], Dinesh Yadav DM[1], Darius Testa Tata[1], Umi Fahmida[3], Babacar Faye[4], Bharati Kulkarni[5], Deepak B. Saxena[6] and Claire Heffernan[1]

[1]Department of Population Health, London School of Hygiene & Tropical Medicine, London, UK

[2]Department of Health Services Research and Policy, London School of Hygiene & Tropical Medicine, London, UK

[3]Southeast Asian Ministers of Education Organization Regional Center for Food and Nutrition (SEAMEO RECFON), Jakarta, Indonesia

[4]Service de Parasitologie-Mycologie- Pédiatrie, Faculté de médecine, Université Cheikh Anta Diop (UCAD), Dakar, Senegal

[5]Clinical Division, ICMR-National Institute of Nutrition, Hyderabad, India

[6]Department of Epidemiology, Indian Institute of Public Health, Gandhinagar, Gujarat, India

Address Correspondence to:

Prof Claire Heffernan, Email: claire.heffernan@lidc.ac.uk

Confidential: For Review Only

Despite concerted efforts, the global community is currently off track in the ambition to reduce the number of stunted children under 5 years of age by 40% by 2025. [1,2] Stunting in children is thought to be a result of adversities in early life with multiple contributors including nutritional inadequacy, environmental insults, and intergenerational transmission of risks. While we know the pathways to stunting are numerous, our understanding of the pathways and their convergence remains a critical block. As such, childhood stunting can be described as a 'mosaic' where there is knowledge of the individual components, but inadequate understanding of the interactions or inter-relationships between the individual elements/drivers comprising the whole. [3] The Action Against Stunting Hub (AASH), a partnership of 18 institutions, funded by the UK Research and Innovation's Global Challenges Research Fund (UKRI GCRF) aims to generate a holistic understanding of the complex drivers of stunting and their interactions to better inform prevention strategies. This article introduces AASH, its interdisciplinary framework and workstreams, the details of which are reported in articles published in this BMJ Paediatrics Open Supplement.

The AASH is an interdisciplinary research project underpinned by 'the Whole Child Approach (WCA). The WCA paradigm originates from the educational sector in USA (United States of America), developed to address the perceived failure of children to thrive.[4] The concept relates children's learning to the core tenets of development referred to as SPECC (Social, Physical, Emotional, Cognitive and Cultural development). The framework was later widened to include the school and community environments in an attempt to better identify and address issues impacting learning at the wider community level. Thus, the WCA radiates the child's microenvironment to the wider context in which the child must navigate. Building on this conceptual frame, AASH adapts the WCA to investigate the complex underpinnings of childhood stunting, shifting focus

Confidential: For Review Only

from the disciplinary silos to building interdisciplinary connections. The AASH uses the WCA to investigate the role of the deep biology of the child (epigenetics, gut health, neurocognition, nutrition, parasitology), the home environment, food environment, water, hygiene, and sanitation (WASH), the educational and care giving environment in stunting. In addition, the WCA elicits and clarifies shared values toward nutrition and stunting at the community level. In this manner, we anticipate this approach will generate a holistic understanding of stunting and support the creation of appropriate and sustainable tools such as decision support tools, predictive markers, and therapeutic targets to address childhood stunting.

The approach is investigated via a combination of observational and intervention studies. The observational study involves prospective pregnancy cohorts in India, Indonesia, and Senegal, where more than 1500 women are recruited during pregnancy with mother-infant pairs followed longitudinally until 24-months post-partum. The intervention trials test the efficacy of egg supplementation during pregnancy and early childhood on child growth and development in Indonesia and India, respectively. Additionally, the impact of synbiotic (a combination of pre- and probiotics) administration during the first 6 months of infancy on child growth and development is currently being assessed in a trial in Senegal. Furthermore, the COVID-19 pandemic and its disruptions to global food supply, earnings, and access to essential healthcare services is projected to contribute to 22 million more stunted children in low- and middle-income countries (LMICs) [5]. The AASH is responding to these emerging exacerbating factors by setting up cohort studies in India to understand the impact of the pandemic and SARS-CoV-2 (severe acute respiratory syndrome – coronavirus 2) infection during pregnancy on food availability and intake, access to healthcare and health services, pregnancy outcomes, growth, and development in early childhood.

Confidential: For Review Only

The workstreams informing the ‘whole child approach’ to stunting include epigenetics, gut health, nutrition, food and home environment, cognition and education and shared values.

Epigenetics is considered a key mechanism through which early life adversities programme both biological functions and long-term health. The epigenetic workstream conducts analyses of both the wider and specific regions of the epigenome in DNA extracts from the saliva samples of fathers, mothers and infants enrolled in the study.

The gut health workstream investigates the relationship between gastrointestinal health and childhood stunting. The gastrointestinal tract facilitates critical physiological functions including transport of nutrients, production of enzymes, hormones, immune functions, which are critical to cell maintenance, promotion of growth and development. As a result, the pathophysiology of the small intestine referred to as EED (environmental enteric dysfunction) is believed to contribute to impaired growth in undernourished children. Further investigation is, however, needed to fully understand the timing, implications, and contributions of enteric dysfunction in childhood stunting, and relationship with other causalities/contributing factors. The gut health workstream uses diverse laboratory methods to investigate gastrointestinal health including biomarkers of EED, intestinal parasites, pathogenic bacteria, and the gut microbiome.

The nutrition workstream investigates the relationship between maternal nutrition, health, and wellbeing, and IYCF (infant and young child feeding) and stunting. Nutrition during pregnancy supports intrauterine growth and development, and IYCF (including breast and complementary feeding) provides critical nutrition during the postnatal period. Inadequate pre and postnatal

Confidential: For Review Only

nutrition is associated with poor birth outcomes, impaired growth, and development in early childhood. However, the contribution of nutrition during these two critical phases of development to childhood stunting and its relationship with other outcomes including gut health, infections, cognition, and epigenetics needs further investigation. The nutrition workstream investigates maternal and infant dietary intake, nutritional status, maternal stress, depression, psychosocial support, fetal growth, and development.

The education and cognition workstream investigate the links between childhood stunting and cognition, care-giving and educational environments. Cognitive deficits, including performance at school, is a well-established consequence of stunting. However, its links to other outcomes including nutrition, gut health, epigenetics, care-giving and early childhood educational environment is not well understood. The workstream uses measures of ECED (early childhood education and development) to profile the learning environment and cognitive development of children. In addition, the workstream is using a case-control study comprising of stunted and non-stunted pre-school aged children (aged between 3.6 to 5.6 years) to further investigate the links between stunting and early child education and development. The ECED assessments are conducted using validated questionnaires including Ox-NDA (oxford neurodevelopment assessment), Inter-NDA (the international fetal and newborn growth consortium for the 21st century neurodevelopment assessment), HOME (home observation for measurement of the environment), MELE (measuring early learning environment), IDELA (international development and early learning assessment) and QTI (qualitative teacher interview*)* and Bayley IV cognition scale.

Confidential: For Review Only

The food systems and home environment workstream investigates the contributions of food and home environment to childhood stunting. Discerning the food environment including food acquisition, factors influencing household food choices and consumption is important in understanding the role of food systems in stunting. Likewise, the home environment including hygiene practices, sources of pathogenic contamination causing food- and water-borne diseases is relevant to providing a better understanding of the role of environmental factors in stunting. The food systems component of the workstream uses a novel food environment tool to investigate the food systems and household food decision making. In addition, value chain analyses are conducted through key informant interviews including farmers, intermediaries, and retailers. Food-based (Optifood) and diet-modelling (Agri-food) software will be used to identify the nutrient adequacy gaps in the diets of mothers and children and recommend areas of interventions to improve nutritional outcomes. The home environment component of the workstream uses a range of approaches including questionnaires, spot checks, biological sampling of household drinking water, foods and domestic surfaces, and direct observation of the home environment to investigate the potential exposure pathways of pathogenic contamination of foods and drinks within the households.

The shared value workstream explores community values regarding childhood stunting. Shared value is defined as implicit or explicit underlying sociocultural beliefs, concepts, and principles. Described in other words as what a group of individuals considers 'valuable, worthwhile and meaningful.' The shared value workstream will investigate the societal beliefs and practices related to stunting and its drivers including breastfeeding practices, dietary intake, antenatal and postnatal clinics attendance, child immunisation, hygiene, and early child education across the study

Confidential: For Review Only

populations. The workstream uses a qualitative data collection tool called WeValue InSitu in local stakeholder group sessions including mothers, fathers, grandmothers, teachers, market vendors and community health workers to allow groups to collectively elicit and articulate their own shared values. This is followed by contextualisation of the communities' lived experiences with childhood stunting through open topic discussions on their roles and behavioural practices relating to early childhood development. The workstream will help advance understanding around the deeply seated societal and community beliefs and behaviours, the impediments and/or affinity towards improving childhood outcomes and help deliver targeted interventions including behavioural change communication.

The operationalisation of the research and subsequent analyses are supported through the creation and implementation of a three-country data repository called the All-Hub Data Repository (AHDR). The repository facilitates the capturing and curation of the research data from the three countries into a central database, and follow-on disciplinary and interdisciplinary analyses of the data. The repository will then be transformed into an interactive, open access data portal, with an imbedded decision support tool, providing unrestricted access to comprehensive data around childhood stunting to support diverse communities including research, programme, and policy communities. Furthermore, the research includes MEL (monitoring, evaluation, and learning) toolkits providing (near) real-time monitoring of the field research activities, tracking its progress, milestones, and sharing learning outcomes.

In conclusion, the interdisciplinary research at AASH presents a new frontier aimed at broadening the understanding of childhood stunting through carefully unpacking its critical predisposing

Confidential: For Review Only

factors and outcomes. This research will move the goalpost from the traditional definition of stunting towards a new typology, and provide researchers, programme and policy developers with the knowledge and tools to guide the global efforts in eradicating childhood stunting and associated developmental delays.

Confidential: For Review Only

REFERENCES

1. de Onis, M., *et al.* The World Health Organization's global target for reducing childhood stunting by 2025: rationale and proposed actions. *Matern Child Nutr* **9 Suppl 2**, 6-26 (2013).
2. Vaivada, T., *et al.* Stunting in childhood: an overview of global burden, trends, determinants, and drivers of decline. *The American Journal of Clinical Nutrition* **112**, 777S-791S (2020).
3. Heffernan, C. Building synergies in development research: is it time for the Mosaic Approach? *Development in Practice* **18**, 686-700 (2008).
4. Lewallen, T.C., Hunt, H., Potts-Datema, W., Zaza, S. & Giles, W. The Whole School, Whole Community, Whole Child model: a new approach for improving educational attainment and healthy development for students. *J Sch Health* **85**, 729-739 (2015).
5. FAO, I., UNICEF, WFP, WHO. *The state of food security and nutrition in the world 2020: transforming food systems for affordable healthy diets*, (Food & Agriculture Org., 2020).

Confidential: For Review Only

CONTRIBUTIONS

MLJ and CH drafted the manuscript and compiled it for publication. All authors reviewed the drafts, contributed to the production of the final draft. All authors approved the final version for submission.

FUNDING STATEMENT

The work was supported by UK Research and Innovation (UKRI) under its Global Challenge Research Fund (GCRF). Funding reference: MR/S01313X/1.

COMPETING INTEREST STATEMENT

Authors declared no competing or conflict of interest.

Confidential: For Review Only

BMJ Paediatrics Open

Unpacking the Intractability of Childhood Stunting: an introduction to the UKRI GCRF Action Against Stunting Hub

Journal:	*BMJ Paediatrics Open*
Manuscript ID	bmjpo-2023-002333.R1
Article Type:	Editorial
Date Submitted by the Author:	27-Nov-2023

Complete List of Authors:	Jobarteh, Modou; London School of Hygiene & Tropical Medicine, Epidemiology & Population Health<br>Conway-Moore, Kaitlin; London School of Hygiene & Tropical Medicine, Health Services Research and Policy<br>Yadav, Dinesh; London School of Hygiene & Tropical Medicine, Department of Population Health<br>Tata, Darius Testa; London School of Hygiene & Tropical Medicine, Department of Population Health<br>Fahmida, Umi; Southeast Asian Ministry of Education Organisation Regional Centre for Food and Nutrition (SEAMEO RECFON)<br>Faye, Babacar; Université Cheikh Anta Diop (UCAD), Faculté de médecine<br>Kulkarni, Bharati; National Institute of Nutrition<br>Saxena, Deepak ; Indian Institute of Public Health Gandhinagar<br>Heffernan, Claire; London School of Hygiene & Tropical Medicine, Department of Population Health

Keywords:	Growth, Epidemiology, Infant, Gastroenterology, Breastfeeding

SCHOLARONE™
Manuscripts

I, the Submitting Author has the right to grant and does grant on behalf of all authors of the Work (as defined in the below author licence), an exclusive licence and/or a non-exclusive licence for contributions from authors who are: i) UK Crown employees; ii) where BMJ has agreed a CC-BY licence shall apply, and/or iii) in accordance with the terms applicable for US Federal Government officers or employees acting as part of their official duties; on a worldwide, perpetual, irrevocable, royalty-free basis to BMJ Publishing Group Ltd ("BMJ") its licensees and where the relevant Journal is co-owned by BMJ to the co-owners of the Journal, to publish the Work in this journal and any other BMJ products and to exploit all rights, as set out in our licence.

The Submitting Author accepts and understands that any supply made under these terms is made by BMJ to the Submitting Author unless you are acting as an employee on behalf of your employer or a postgraduate student of an affiliated institution which is paying any applicable article publishing charge ("APC") for Open Access articles. Where the Submitting Author wishes to make the Work available on an Open Access basis (and intends to pay the relevant APC), the terms of reuse of such Open Access shall be governed by a Creative Commons licence – details of these licences and which Creative Commons licence will apply to this Work are set out in our licence referred to above.

Other than as permitted in any relevant BMJ Author's Self Archiving Policies, I confirm this Work has not been accepted for publication elsewhere, is not being considered for publication elsewhere and does not duplicate material already published. I confirm all authors consent to publication of this Work and authorise the granting of this licence.

Confidential: For Review Only

Unpacking the Intractability of Childhood Stunting: an introduction to the UKRI GCRF Action Against Stunting Hub

Modou Lamin Jobarteh[1], Kaitlin Conway-Moore[2], Dinesh Yadav DM[1], Darius Testa Tata[1], Umi Fahmida[3], Babacar Faye[4], Bharati Kulkarni[5], Deepak B. Saxena[6] and Claire Heffernan[1]

[1]Department of Population Health, London School of Hygiene & Tropical Medicine, London, UK

[2]Department of Health Services Research and Policy, London School of Hygiene & Tropical Medicine, London, UK

[3]Southeast Asian Ministers of Education Organization Regional Center for Food and Nutrition (SEAMEO RECFON), Jakarta, Indonesia

[4]Service de Parasitologie-Mycologie- Pédiatrie, Faculté de médecine, Université Cheikh Anta Diop (UCAD), Dakar, Senegal

[5]Clinical Division, ICMR-National Institute of Nutrition, Hyderabad, India

[6]Department of Epidemiology, Indian Institute of Public Health, Gandhinagar, Gujarat, India

Address Correspondence to:

Prof Claire Heffernan, Email: claire.heffernan@lidc.ac.uk

Confidential: For Review Only

Despite concerted efforts, the global community is off-track in its ambition to reduce the number of stunted children under 5 years by 40% by 2025 [1, 2]. Stunting in children is thought to be a result of adversities in early life with multiple contributors including inadequate nutritional intake, environmental insults, and inter-generational transmission of risk. While we know the pathways to stunting are numerous, our understanding of the convergence of these pathways remains a critical block. As such, childhood stunting can be described as a 'mosaic' where there is knowledge of the individual components, but inadequate understanding of the interactions or inter-relationships between the individual elements/drivers comprising the whole [3]. In addition, the current definition of stunting is based on measurement of child length, calculated as Length-for-Age-Z score (LAZ) 2 standard deviations below the median of the WHO growth standard. Whilst this is important in adopting a universally accepted standard definition, the approach is rather too simplistic. The definition does not take into consideration the profoundly complex pathophysiology of stunting and its far-reaching consequences on growth and development, future health and wellbeing of the affected children, communities, and nations. Accordingly, research on stunting tends to focus on a single outcome or at best, parts of a system, with inadequate holistic exploration of underlying interconnections. Indeed, by defining childhood stunting as a single outcome, it may be argued that we have fostered a lack of understanding of the pathway each child has been on to reach such an outcome. The Action Against Stunting Hub (AASH), a partnership of 18 institutions, funded by the UK Research and Innovation's Global Challenges Research Fund (UKRI GCRF) aims to generate a holistic understanding of the complex drivers of stunting and their interactions to better inform prevention and treatment strategies. This article introduces AASH, the related workstreams and interdisciplinary synergies.

Confidential: For Review Only

The AASH's approach to broaden the understanding of child stunting was inspired by 'the development niche' paradigm developed by Harkness and Super to investigate child development [4]. According to the authors, the developmental niche is comprised of three core elements: a) the physical and social environment underpinning a child's daily life, b) the dominant cultural norms regarding childcare/rearing, and c) the psychological make-up of parents and caretakers. The AASH then adapted the 'developmental niche paradigm' with the addition of a fourth element, the child's basic biology and reframed it as the 'bio-development' niche. The bio-development niche is operationalised using 'the Whole Child Approach (WCA). The WCA investigates the role of the deep biology (epigenetics, gut health, neurocognition, nutrition, parasitology), the home environment, food environment, water, hygiene, and sanitation (WASH), the educational and care giving environment in childhood stunting. The WCA also elicits and clarifies shared values toward nutrition and stunting at the community level (**Figure 1**). Thus, the WCA is utilised to investigate the complex underpinnings of childhood stunting, shifting focus from the disciplinary silos to building interdisciplinary connections. In this manner, we anticipate this approach will generate a holistic understanding of stunting and support the creation of appropriate tools such as decision support tools, predictive markers, and therapeutic targets to address childhood stunting.

The WCA is investigated via a combination of observational and intervention studies. The observational study involves prospective pregnancy cohorts in India, Indonesia, and Senegal, where women are recruited during pregnancy with mother-infant pairs followed longitudinally until 24-months post-partum. The intervention trials test the efficacy of egg supplementation during pregnancy and early childhood on child growth and development in Indonesia and India, respectively. Additionally, the impact of synbiotic (a combination of pre- and probiotics)

Confidential: For Review Only

administration during the first 6 months of infancy on child growth and development is currently being assessed in a trial in Senegal. Furthermore, the COVID-19 pandemic and its disruptions to the global food supply, earnings, and access to essential healthcare services is projected increase the number stunted children in low- and middle-income countries (LMICs) by an estimated 22 million more children [5]. The AASH is responding to these emerging exacerbating factors by setting up cohort studies in India to understand the impact of the pandemic and SARS-CoV-2 (severe acute respiratory syndrome – coronavirus 2) infection during pregnancy on food availability and intake, access to healthcare and health services, pregnancy outcomes, growth, and development in early childhood.

The workstreams informing the 'whole child approach' to stunting are epigenetics, gut health, nutrition, food and home environment, cognition and education, and shared values. The detailed protocols underpinning each workstream are published in this BMJ Paediatrics Open Supplement. Briefly, the epigenetic workstream conducts analyses of both the wider and specific regions of the epigenome in DNA extracts from the saliva samples of fathers, mothers and infants enrolled in the study. The gut health workstream investigates the relationship between gastrointestinal health and childhood stunting with the aim to better understand the timing, implications, and contributions of enteric dysfunctions in childhood stunting. The nutrition workstream investigates the role of maternal nutrition, health, and wellbeing, and IYCF (infant and young child feeding) on stunting. This workstream also investigates maternal stress, depression, psychosocial support on fetal and infant growth and development. The education and cognition workstream investigate the links between childhood stunting and cognition, caregiving, and the wider educational environment. The workstream uses measures of ECED (early childhood education and development) to profile

Confidential: For Review Only

the early development of children. The food systems and home environment workstream investigates the contributions of food and home environment to childhood stunting. The shared value workstream explores community values regarding childhood stunting. Shared value is defined as implicit or explicit underlying sociocultural beliefs, concepts, and principles. The workstream will investigate the societal beliefs and practices related to stunting and its drivers.

Whilst unravelling the roles of these individual components is important, the interdisciplinary connections, interactions, and synergies between the workstreams moves the discourse beyond disciplinary boundaries into a novel, holistic understanding of the complex underpinnings of childhood stunting. The AASH is developing an interdisciplinary aid within its All-Hub Data Repository (AHDR) to support exploration of the interdisciplinary interactions. The repository also facilitates the capturing and curation of research data from the three countries into a central database with a range of bespoke tools including a decision support tool. Equally importantly, the research includes a MEL (Monitoring, Evaluation, and Learning) toolkit to support monitoring of the field research activities, tracking its progress, milestones and share learning outcomes in (near) real-time.

In conclusion, the interdisciplinary research at AASH presents a new frontier aimed at moving the dial away individual exposures to broadening the understanding of childhood stunting by carefully unpacking its critical predisposing factors and outcomes. In this manner, we believe the AASH will provide researchers, programme and policy developers with the knowledge and tools to guide the global efforts in eradicating childhood stunting and its associated developmental delays.

Confidential: For Review Only

REFERENCES

1. de Onis M, Dewey KG, Borghi E, Onyango AW, Blössner M, Daelmans B, Piwoz E, Branca F: **The World Health Organization's global target for reducing childhood stunting by 2025: rationale and proposed actions.** *Matern Child Nutr* 2013, **9 Suppl 2:**6-26.
2. Vaivada T, Akseer N, Akseer S, Somaskandan A, Stefopulos M, Bhutta ZA: **Stunting in childhood: an overview of global burden, trends, determinants, and drivers of decline.** *The American Journal of Clinical Nutrition* 2020, **112:**777S-791S.
3. Heffernan C: **Building synergies in development research: is it time for the Mosaic Approach?** *Development in Practice* 2008, **18:**686-700.
4. Super CM, Harkness S: **The Developmental Niche: A Conceptualization at the Interface of Child and Culture.** *International Journal of Behavioral Development* 1986, **9:**545-569.
5. FAO I, UNICEF, WFP, WHO: *The state of food security and nutrition in the world 2020: transforming food systems for affordable healthy diets.* Food & Agriculture Org.; 2020.

Confidential: For Review Only

CONTRIBUTIONS

MLJ and CH drafted the manuscript and compiled it for publication. KCM, DYDM, DTT, UF, BF, BK and DBS reviewed the drafts and provided valuable inputs in writing the manuscript. All authors contributed to the production of the final draft and approved the final version for submission.

FUNDING STATEMENT

The work was supported by UK Research and Innovation (UKRI) under its Global Challenge Research Fund (GCRF). Funding reference: MR/S01313X/1.

COMPETING INTEREST STATEMENT

Authors declared no competing or conflict of interest.

Confidential: For Review Only

FIGURE LEGEND

Figure 1: UKRI GCRF Action Against Stunting Hub 'Whole Child Approach' to investigate childhood stunting.

A child-centric conceptual framework focusing on the 'whole' child, instead of the disparate drivers, to provide a holistic understanding of the drivers and outcomes associated with stunting. The approach investigates the role of the home environment, food systems, dietary intake, water, hygiene, and sanitation (WASH), shared values, neurocognition and education environments, gut health, microbiome, nutrition, parasitology, and epigenetics in childhood stunting.

Confidential: For Review Only

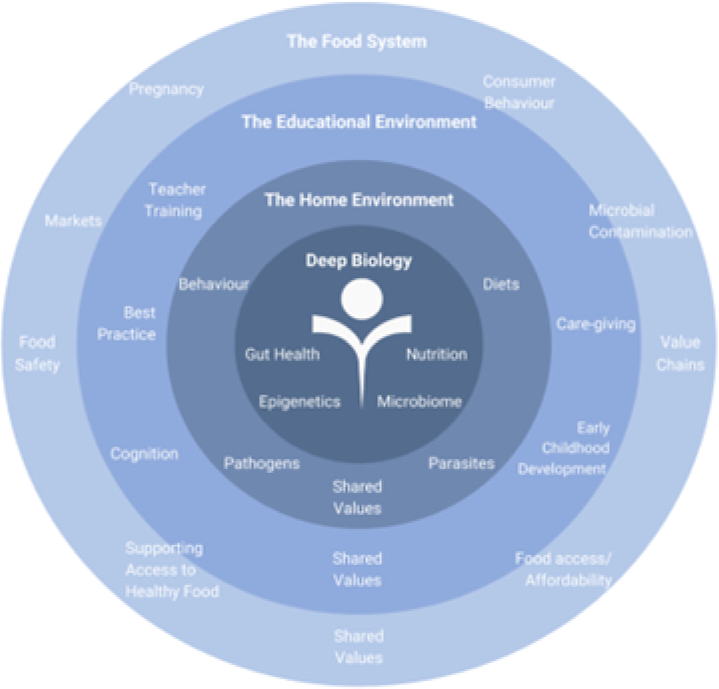

UKRI GCRF Action Against Stunting Hub 'Whole Child Approach' to investigate childhood stunting.

A child-centric conceptual framework focusing on the 'whole' child, instead of the disparate drivers, to provide a holistic understanding of the drivers and outcomes associated with stunting. The approach investigates the role of the home environment, food systems, dietary intake, water, hygiene, and sanitation (WASH), shared values, neurocognition and education environments, gut health, microbiome, nutrition, parasitology, and epigenetics in childhood stunting.

331x328mm (28 x 28 DPI)
