# Supplementary material for: Unpacking the intractability of childhood stunting: an introduction to the UKRI GCRF Action Against Stunting Hub
Source: BMJ Paediatr Open. 2024 Mar 22;8(Suppl 1):e002333. doi: 10.1136/bmjpo-2023-002333 (PMC10961497; doi:10.1136/bmjpo-2023-002333)
Supplement: Reviewer comments [file bmjpo-2023-002333.reviewer_comments.pdf]

PEER REVIEW HISTORY

BMJ Paediatrics Open publishes all reviews undertaken for accepted manuscripts. Reviewers are asked to complete a checklist review form and are provided with free text boxes to elaborate on their assessment. These free text comments are reproduced below.

ARTICLE DETAILS

**TITLE (PROVISIONAL)**	Unpacking the Intractability of Childhood Stunting: an introduction to the UKRI GCRF Action Against Stunting Hub
**AUTHORS**	Jobarteh, Modou Lamin<br>Conway-Moore, Kaitlin<br>Yadav, Dinesh<br>Tata, Darius Testa<br>Fahmida, Umi<br>Faye, Babacar<br>Kulkarni, Bharati<br>Saxena, Deepak<br>Heffernan, Claire

This article was not externally reviewed at BMJ Paediatrics Open. Protocols that have been independently assessed prior to submission to BMJ Paediatrics Open are usually fast-tracked to publication on the grounds that further substantial changes will not be possible. This independent assessment will usually be external, independent review for both a substantial grant award from a non-commercial or government funder and ethics approval.
